# Daily light integral and/or photoperiod during the young plant and finishing stages influence floral initiation and quality of witchgrass and marigold cut flowers

**DOI:** 10.3389/fpls.2022.956157

**Published:** 2022-09-14

**Authors:** Caleb E. Spall, Roberto G. Lopez

**Affiliations:** Department of Horticulture, Michigan State University, East Lansing, MI, United States

**Keywords:** light-emitting diodes, *Panicum*, specialty cut flowers, supplemental lighting, *Tagetes*, young plants

## Abstract

To produce consistent and high-quality specialty cut flowers throughout the year, growers in temperate climates must utilize controlled environment greenhouses. Research-based information on photoperiod management and supplemental lighting for specialty cut flowers is limiting. Therefore, our objectives were (1) to determine the effect of photoperiod during the young-plant and finishing stages on floral initiation and morphology of witchgrass ‘Frosted Explosion’ (*Panicum capillare*) and marigold ‘Xochi’ (*Tagetes erecta*) and (2) to quantify the effect of daily light integral (DLI) on floral initiation and morphology of witchgrass during the finishing stage. Seeds of marigold and multi-seed pellets of witchgrass were sown and placed under 9-, 11- (marigold only), 12-, 13-, 14-, 15-, 16-, 18-, or 24-h photoperiods or a 9-h short day with a 4-h night interruption (NI) from 2200 to 0200 h. Plugs were distributed among 10-, 11-, 12-, 13-, 14-, 15-, or 16-h photoperiods or a 4-h NI, for finishing. Witchgrass was finished under a very low or moderate DLI of ≈3 or 10 mol^⋅^m^–2⋅^d^–1^, respectively, while marigold was finished under a DLI of ≈10 mol^⋅^m^–2⋅^d^–1^. Marigold grown under a photoperiod ≥ 11 h or a 4-h NI during the young-plant stage and finished under an 11- or 12-h photoperiod had thick stems and consistently met the marketable stem length of ≥ 65 cm. Up to 29% and 107% more stems were harvestable under 11- and 12-h finishing photoperiods, respectively, compared to a 10-h finishing photoperiod. Marigold visible buds were delayed, and stems were not harvestable under photoperiods ≥ 13 h or a 4-h NI after 8 weeks. Young witchgrass plants grown under a photoperiod between 14- and 24-h or a 4-h NI and finished under photoperiods ≥ 14 h or a 4-h NI, and at least a moderate DLI, were reliably harvestable (≥ 50 cm long with a fully developed panicle). Witchgrass finished under day lengths < 13 h (rep. 1) or < 14 h (rep. 2) flowered prematurely and were roughly one-sixth the length of harvestable stems at an open flower. All witchgrass stems grown under a very low DLI were shorter and thinner than those grown under a moderate DLI, and none were harvestable. Therefore, we recommend growing marigold ‘Xochi’ young plants under a photoperiod ≥ 11 h or a 4-h NI and finishing under a 12-h photoperiod. Additionally, witchgrass ‘Frosted Explosion’ young plants should be grown under a photoperiod ≥ 14 h or a 4-h NI and finished under photoperiods ≥ 14 h or a 4-h NI to prevent premature flowering. Witchgrass and marigold cut flowers should be finished under a DLI of ≥ 10 mol^⋅^m^–2⋅^d^–1^ for consistent production of high-quality stems.

## Introduction

Year-round demand for locally sourced specialty cut flowers continues to increase in the United States [Faust and Dole, 2021; Produce Marketing Association and Food Marketing Institute [PMAFMI], 2016]. From 2015 to 2018, the number of domestic cut flower producers with annual sales ≥ $100,000 increased by 20%, and producers reported a wholesale value of $374 million in 2018 [US Department of Agriculture [USDA]., 2019]. Of this, California accounted for $288 million (77%) of domestic production at least partly because of the coastal climates of its central and southern counties (Carman, 2007). However, demand persists across the nation, and growers in northern latitudes cannot produce specialty cut flowers outdoors year-round due to low temperatures and solar radiation during the winter and early spring. Thus, controlled-environment greenhouses must be utilized to produce high-quality specialty cut flowers year-round.

Many varieties of specialty cut flowers are categorized as short-day plants (SDPs), including marigold (*Tagetes erecta*), celosia (*Celosia* spp.), and zinnia (*Zinnia elegans*) (Craig and Runkle, 2013; Dole, 2015). Young plants with a short day (SD) flowering response may flower prematurely if grown during periods with natural SDs, resulting in short, unmarketable stems (Dole and Warner, 2017). Therefore, photoperiodic lighting techniques such as low-intensity day extension (DE), night interruption (NI), or high-intensity cyclic lighting can be utilized to create long days during the beginning of the production cycle (Meng and Runkle, 2016), ensuring that plants do not flower prematurely, and thereby preventing inferior cut flower quality (Currey et al., 2013).

Preventing premature flowering through photoperiod manipulation may also reduce the need for plant growth regulator applications. Once flower initiation has occurred, it is rarely possible to revert plants to a vegetative state by placing them under non-inductive photoperiods (Runkle, 2008) or by removing flower buds. Thus, flower-aborting plant growth regulators such as ethephon must be applied, and multiple applications may be necessary over the duration of the crop cycle (Styer, 2002). Additionally, such plant growth regulators can inhibit internode elongation and suppress apical dominance (Runkle, 2013). Therefore, it is recommended that cut flowers be grown under non-inductive photoperiods for several weeks before flower initiation (Porat et al., 1995; Dole and Warner, 2017).

Limited research-based information detailing photoperiodic lighting applications for greenhouse-grown SDP cut flowers exists. Blacquière (2002) reported that a low-intensity 2-h NI was effective at inhibiting the flowering of chrysanthemum ‘White Reagen’ and ‘Majoor Bosshardt’ (*Chrysanthemum* × *morifolium* Kitamura) by 28 and 30 d, respectively. Furthermore, Park and Jeong (2019) demonstrated the efficacy of a 16-h photoperiod and 4-h NI of various light qualities at inhibiting the flowering of chrysanthemum ‘Gaya Yellow’ for the duration of the study (46 d) when applied at intensities of 180 and 10 μmol^⋅^m^–2⋅^s^–1^, respectively, whereas SD conditions promoted flower bud initiation after 21 d. In addition, LDs and NIs provided by red (R; 600–700 nm), white (W; 400–700 nm), and far-red (FR; 700–800 nm) radiation resulted in crops that were 6–8 cm taller than those grown under SDs (Park and Jeong, 2019). In a separate study, pinched celosia ‘Rocket’ (*Celosia argentea* var. *plumosa*) grown under a 16-h photoperiod for 3 weeks, and then an 8-h photoperiod for 29 d, had four times as many stems per plant and were ≈183% taller than pinched plants grown under continuous SDs for 50 d (Porat et al., 1995).

In addition to regulating photoperiod, growers must maintain sufficient radiation intensities through the use of supplemental lighting (SL) when solar radiation intensities are low (Wollaeger and Runkle, 2014) to consistently produce high-quality cut flowers. This is especially important in northern latitudes as the outdoor daily light integral (DLI) can fall to 5 to 10 mol^⋅^m^–2⋅^d^–1^ during the winter and early spring (Korczynski et al., 2002), and can drop further to < 5 mol^⋅^m^–2⋅^d^–1^ in greenhouses due to reflection of incoming radiation from greenhouse glazing and shading from the greenhouse superstructure (Lopez and Runkle, 2008). Increasing the DLI with SL to produce greenhouse crops other than specialty cut flowers is well-documented. Generally, a moderate to high DLI (e.g., 8 to 12 mol^⋅^m^–2⋅^d^–1^) during the young-plant and finishing stages elicits favorable growth responses, including a reduction in time to flower and an increase in biomass and finished plant quality (Faust et al., 2005; Pramuk and Runkle, 2005; Owen et al., 2018). Research documenting the use of SL to increase the DLI during specialty cut flower production is limited. By reducing the time to flower, growers gain the potential for more production cycles per season, and thus, the potential for increased annual income. For instance, godetia (*Clarkia amoena*) ‘Satin White,’ ‘Salmon,’ ‘Rose Pink,’ and ‘Red’ flowered ≈41, 94, 98, and 114 d faster, respectively, when grown under SL providing 79 μmol^⋅^m^–2⋅^s^–1^ from 1800 to 2400 h in comparison to those grown without SL in autumn (Anderson, 1993). Although the finished stem length of godetia was 19% to 33% shorter when grown under SL compared to those grown without SL (Anderson, 1993), the finished stems were still of sufficient length for sale. Similarly, time to flower and height of oriental lily (*Lilium* spp.) ‘Laura Lee’ were reduced by an average of 21 d and 20%, respectively, when grown under SL providing 60 μmol^⋅^m^–2⋅^s^–1^ for 5 h per day, compared to those grown without SL (Treder, 2003).

High DLIs also have the potential to increase harvestable cut flower yields (Dole and Warner, 2017). Stem yield of gerbera ‘Estelle’ and ‘Ximena’ (*Gerbera* × *cantabrigensis*) increased by 13 and 10 stems, respectively, when grown under a DLI of 6.5 mol^⋅^m^–2⋅^d^–1^ in comparison to 3.2 mol^⋅^m^–2⋅^d^–1^ (Autio, 2000). The stem yield of gerbera ‘Panama’ increased by 40% as the DLI increased from 5.3 to 11.3 mol^⋅^m^–2⋅^d^–1^ (Llewellyn et al., 2020). Increased cut flower yield under higher DLIs is partly due to increased branching for some varieties. Lim et al. (2022) reported that mountain spike speedwell (*Veronica rotunda* var. *subintegra*) and long-leaf spike speedwell (*Veronica longifolia*) had 331% and 308% more branches when grown under a DLI of 18.3 mol^⋅^m^–2⋅^d^–1^ compared to 6.6 mol^⋅^m^–2⋅^d^–1^ for 12 weeks.

Additional research quantifying the influence of photoperiod and DLI on the growth and development of greenhouse-grown specialty cut flowers is needed for growers in northern latitudes. Therefore, the objectives of this study were to (1) determine how various photoperiods during the young-plant and finishing stages interact to influence floral initiation and morphology of witchgrass ‘Frosted Explosion’ (*Panicum capillare*) and marigold ‘Xochi’ (*Tagetes erecta*) and (2) quantify how DLI influences floral initiation and morphology of witchgrass during the finishing stage. We hypothesized that both witchgrass and marigold would exhibit a facultative SD response, characterized by delayed flowering and longer stem lengths as the young-plant (seedling stage) and finishing (remainder of the crop cycle after transplant) photoperiods increased. We also hypothesized that witchgrass grown under a moderate DLI would be of higher quality, although shorter, compared to those grown under a very low DLI.

## Materials and methods

### Young plant material, culture, and lighting treatments

Multi-seed pellets of witchgrass ‘Frosted Explosion’ (PanAmerican Seed, West Chicago, IL, United States) and seeds of marigold ‘Xochi’ (PanAmerican Seed) were sown in 288-cell (7 mL individual volume) trays by a commercial propagator (Raker-Roberta’s Young Plants, Litchfield, MI, United States). These genera and varieties were selected as they were recent introductions with reports of premature flowering. Nine plug trays of witchgrass and 10 plug trays of marigold were received on 15 September 2020 (Rep. 1) and 8 September 2021 (Rep. 2), 1 day after sowing. Each tray was divided into two blocks of 144 cells. The blocks were randomly and equally distributed in a greenhouse at the Michigan State University (East Lansing, MI; lat. 43°N) under various photoperiodic treatments. Photoperiodic treatments consisted of a 9-h SD (0800 to 1700 h) or a 9-h SD extended with four R + W + FR light-emitting diode (LED) lamps (Arize Greenhouse Pro; General Electric, Boston, MA, United States) on each bench to create 9-, 11- (marigold only), 12-, 13-, 14-, 15-, 16-, 18-, or 24-h photoperiods or a 4-h NI from 2200 to 0200 h. Each LED lamp was covered with multiple layers of aluminum wire mesh (General purpose aluminum; New York Wire, Grand Island, NY, United States) to achieve an average total photon flux density (TPFD) of 2 to 3 μmol^⋅^m^–2⋅^s^–1^ between 400 and 800 nm. The 100-nm waveband ratios (%) emitted by the LED lamps, defined by their B (400–500 nm), green (G; 500–600 nm), R, and FR photon flux densities (PFDs), were 6:19:45:30.

### Young plant greenhouse environment

Young plants were grown in a glass-glazed greenhouse with exhaust fans, evaporative-pad cooling, radiant hot-water heating, and SL controlled by an environmental control system (Priva Integro 725; Priva North America, Vineland Station, ON, Canada). High-intensity LED fixtures (Philips GP-TOPlight DRW-MB; Koninklijke Philips N.V., Eindhoven, Netherlands) provided a supplemental photosynthetic photon flux density (PPFD) of 120 ± 10 μmol^⋅^m^–2⋅^s^–1^ [as measured with a quantum sensor (LI-190R; LI-COR Biosciences, Lincoln, NE, United States)] when the ambient PPFD dropped below ≈400 μmol^⋅^m^–2⋅^s^–1^ between 0800 and 1700 h. On each bench, a line quantum sensor (LI-191R, LI-COR, Lincoln, NE, United States) or a quantum sensor (LI-190R, LI-COR, Lincoln, NE, United States) positioned horizontally at plant height measured PPFD every 10 s and a datalogger (CR1000; Campbell Scientific, Logan, UT, United States) recorded hourly averages. The actual DLIs during the young-plant stages of the two replications of the experiment were 10.3 to 11.7 mol^⋅^m^–2⋅^d^–1^ (Table 1). The 100-nm waveband ratios (%) emitted by the LED fixtures, defined by their B, G, and R photon flux densities, were 10:5:85.

**TABLE 1:** Actual daily light integral (DLI) [mean ± SD (mol^⋅^m^–2⋅^d^–1^)], air average daily temperature (ADT), day temperature, and night temperature [mean ± SD (°C)] throughout the duration of the witchgrass and marigold young-plant stage for reps. 1 and 2.

Photoperiod (h)	DLI (mol^⋅^m^–2⋅^d^–1^)	ADT (°C)	Day (°C)	Night (°C)
*Rep. 1*				
9	–*^z^*	20.1 ± 1.0	21.6 ± 2.5	18.6 ± 2.5
11	–*^z^*	20.1 ± 1.0	21.6 ± 2.5	18.6 ± 2.5
12	–*^z^*	–*^z^*	–*^z^*	–*^z^*
13	–*^z^*	–*^z^*	–*^z^*	–*^z^*
14	10.6 ± 3.6	21.5 ± 1.0	22.8 ± 2.1	20.1 ± 2.0
15	11.1 ± 1.9	–*^z^*	–*^z^*	–*^z^*
16	10.9 ± 3.7	21.4 ± 1.1	23.1 ± 3.0	19.7 ± 3.1
18	10.5 ± 3.6	21.2 ± 0.9	22.8 ± 2.7	19.5 ± 2.8
24	10.7 ± 3.8	22.0 ± 1.3	23.7 ± 3.9	20.3 ± 4.3
4-h NI	10.3 ± 3.2	21.0 ± 1.0	22.0 ± 1.8	19.9 ± 1.5
*Rep. 2*				
9	10.5 ± 5.4	21.1 ± 2.0	21.5 ± 4.1	20.6 ± 3.5
11	–*^z^*	20.4 ± 2.1	21.0 ± 4.1	19.7 ± 3.1
12	11.3 ± 6.2	20.6 ± 2.1	21.3 ± 4.3	19.8 ± 3.1
13	10.6 ± 4.9	20.5 ± 2.3	21.0 ± 4.1	19.8 ± 3.2
14	–*^z^*	20.4 ± 2.2	20.9 ± 4.0	19.8 ± 3.1
15	10.4 ± 6.7	20.9 ± 2.5	21.4 ± 4.4	20.4 ± 3.7
16	11.7 ± 6.4	20.4 ± 2.3	20.9 ± 4.1	19.8 ± 3.2
18	10.8 ± 5.6	19.8 ± 2.1	19.8 ± 3.5	19.9 ± 3.3
24	10.7 ± 5.9	20.5 ± 2.0	20.8 ± 3.7	20.2 ± 3.2
4-h NI	11.6 ± 5.9	21.1 ± 2.1	22.0 ± 5.0	20.1 ± 3.3

^z^No data recorded.

The greenhouse air average daily temperature (ADT) set point was 20°C (12 h day/12 h night at 22/18°C), with daytime and nighttime temperatures maintained from 0500 to 1700 h and 1700 to 0500 h, respectively. An aspirated thermocouple [36-gauge (0.127 mm diameter) type E, Omega Engineering, Stamford, CT] positioned in the middle of each bench measured the air temperature at plant height every 10 s, and the data logger recorded hourly means. The data logger controlled a 1,500-W electric heater underneath each bench to provide supplemental heat when the nighttime temperature was < 19.8°C. The actual air ADT and average day and night temperature at plant height of each treatment during the young-plant stages are provided in Table 1.

Young plants were irrigated as needed with MSU Plug Special [13N-2.2P-10.8K water-soluble fertilizer containing (mg^⋅^L^–1^) 61 nitrogen, 10 phosphorus, 50 potassium, 28.1 calcium, 4.7 magnesium, 1.3 iron, 0.6 manganese, 0.6 zinc, 0.6 copper, 0.4 boron, and 0.1 molybdenum; (GreenCare Fertilizers Inc., Kankakee, IL, United States)] blended with reverse-osmosis water and applied with a mist nozzle (Super Fine Fogg-It Nozzle; Fogg-It Nozzle Co., Inc., Belmont, CA, United States).

### Finished plant lighting treatments, greenhouse environment, and culture

The same high-intensity LED fixtures described above provided a supplemental PPFD of 120 ± 10 μmol^⋅^m^–2⋅^s^–1^ (as measured with a quantum sensor) from 0800 to 1700 h. Additionally, a combination of whitewash applied to the exterior of the greenhouse (KoolRay Classic Liquid Shade, Continental Products, Euclid, OH, United States) and shade cloth surrounding benches (Harmony 5120 OE, Ludvig Svensson Inc., Charlotte, NC, United States) was utilized to create DLIs of ≈3 (very low) and ≈10 mol^⋅^m^–2⋅^d^–1^ (moderate). The actual DLIs on each bench during the finishing stages of the two replications of the experiment were calculated and are provided in Tables 2, 3. For both witchgrass and marigold, photoperiods of 10-, 11-, 12-, 13-, 14-, 15-, or 16-h, or a 9-h SD with a 4-h NI from 2200 to 0200 h, were maintained with the same methods and equipment described in the section “young plant greenhouse environment”. Greenhouse temperature set points during the finishing stage were identical to those in the young-plant stage. The actual air ADT and average day and night temperature at plant height of each treatment during the finishing stages are provided in Tables 2, 3.

**TABLE 2:** Actual DLIs [mean ± SD (mol^⋅^m^–2⋅^d^–1^)], air ADTs, mean day temperature, and mean night temperature [mean ± SD (°C)] throughout the duration of the witchgrass finishing stage for reps. 1 and 2.

		Moderate DLI				Very low DLI		
Photoperiod (h)	DLI (mol^⋅^m^–2⋅^d^–1^)	ADT (°C)	Day (°C)	Night (°C)	DLI (mol^⋅^m^–2⋅^d^–1^)	ADT (°C)	Day (°C)	Night (°C)
*Rep. 1*								
10	10.0 ± 4.8	19.7 ± 1.4	22.1 ± 3.3	17.2 ± 3.2	2.9 ± 1.0	20.3 ± 1.2	22.3 ± 2.4	18.3 ± 2.4
11	–*^z^*	19.9 ± 1.2	22.0 ± 2.7	17.9 ± 2.5	2.7 ± 1.0	20.6 ± 0.9	22.7 ± 2.3	18.5 ± 2.1
12	10.1 ± 4.8	20.9 ± 1.2	23.3 ± 3.0	18.5 ± 2.9	2.7 ± 0.9	20.3 ± 1.1	22.1 ± 2.2	18.5 ± 2.2
13	–*^z^*	–*^z^*	–*^z^*	–*^z^*	2.9 ± 0.9	20.4 ± 1.0	22.2 ± 2.3	18.5 ± 2.1
14	10.6 ± 4.9	20.8 ± 1.3	23.0 ± 3.2	18.6 ± 2.5	–*^z^*	19.9 ± 1.3	21.9 ± 2.6	17.9 ± 2.7
15	11.6 ± 4.7	–*^z^*	–*^z^*	–*^z^*	3.1 ± 0.9	20.7 ± 0.9	22.6 ± 2.6	18.8 ± 2.2
16	10.8 ± 4.4	20.3 ± 1.0	22.2 ± 2.8	18.4 ± 2.4	2.9 ± 1.1	20.6 ± 1.0	22.6 ± 2.2	18.6 ± 2.0
4-h NI	10.3 ± 3.9	20.4 ± 0.9	21.8 ± 2.2	19.0 ± 1.7	–*^z^*	20.3 ± 1.4	22.4 ± 2.6	18.1 ± 2.6
*Rep. 2*								
10	10.0 ± 3.5	20.1 ± 1.8	22.2 ± 3.1	18.0 ± 3.2	3.0 ± 1.6	19.7 ± 1.6	21.8 ± 2.6	17.6 ± 3.2
11	9.8 ± 3.1	20.1 ± 1.3	22.2 ± 2.7	18.0 ± 2.8	3.6 ± 1.8	19.6 ± 1.2	22.0 ± 2.5	17.3 ± 2.7
12	10.0 ± 3.2	20.3 ± 1.1	22.3 ± 2.7	18.4 ± 2.5	3.4 ± 1.8	19.8 ± 1.9	21.9 ± 3.0	17.7 ± 3.2
13	–*^z^*	19.1 ± 2.0	20.6 ± 3.8	17.1 ± 3.7	2.9 ± 1.8	19.6 ± 1.2	21.9 ± 2.5	17.3 ± 2.7
14	10.1 ± 3.0	19.1 ± 1.9	20.6 ± 3.8	17.3 ± 3.4	3.1 ± 2.0	19.4 ± 1.8	21.6 ± 2.9	17.2 ± 3.5
15	9.8 ± 2.9	20.4 ± 1.6	22.4 ± 2.7	18.4 ± 3.2	3.2 ± 1.7	19.5 ± 1.4	21.6 ± 2.5	17.3 ± 2.9
16	10.2 ± 4.2	–*^z^*	–*^z^*	–*^z^*	2.9 ± 1.4	19.5 ± 1.6	21.6 ± 2.5	17.4 ± 3.2
4-h NI	–*^z^*	18.9 ± 1.9	20.9 ± 2.9	16.8 ± 3.4	3.6 ± 1.9	19.2 ± 1.4	21.5 ± 2.5	17.0 ± 3.0

^z^No data recorded.

**TABLE 3:** Actual DLIs [mean ± SD (mol^⋅^m^–2⋅^d^–1^)], air ADTs, mean day temperature, and mean night temperature [mean ± SD (°C)] throughout the duration of the marigold finishing stage for reps. 1 and 2.

Photoperiod (h)	DLI (mol^⋅^m^–2⋅^d^–1^)	ADT (°C)	Day (°C)	Night (°C)
*Rep. 1*				
10	10.6 ± 4.2	19.5 ± 1.4	22.1 ± 3.5	17.0 ± 3.3
11	–*^z^*	20.1 ± 1.3	22.1 ± 2.7	18.0 ± 2.6
12	10.6 ± 4.8	21.0 ± 1.2	23.4 ± 3.0	18.7 ± 2.9
13	–*^z^*	–*^z^*	–*^z^*	–*^z^*
14	11.0 ± 4.8	21.0 ± 1.3	23.2 ± 3.2	18.8 ± 2.5
15	11.9 ± 4.6	–*^z^*	–*^z^*	–*^z^*
16	11.2 ± 4.4	20.4 ± 1.1	22.3 ± 2.9	18.4 ± 2.5
4-h NI	10.8 ± 3.8	20.5 ± 0.9	21.9 ± 2.2	19.1 ± 1.7
*Rep. 2*				
10	9.8 ± 2.0	19.8 ± 1.9	22.0 ± 3.3	17.7 ± 3.5
11	9.0 ± 1.7	20.4 ± 1.3	22.3 ± 2.6	18.6 ± 2.7
12	10.0 ± 2.1	20.4 ± 1.2	22.3 ± 2.8	18.6 ± 2.7
13	9.8 ± 1.5	19.3 ± 2.1	20.6 ± 4.1	17.6 ± 4.0
14	10.1 ± 2.6	19.4 ± 2.0	20.5 ± 4.1	17.9 ± 3.6
15	10.0 ± 1.7	20.8 ± 1.5	22.7 ± 2.7	18.9 ± 3.2
16	10.4 ± 2.9	–*^z^*	–*^z^*	–*^z^*
4-h NI	–*^z^*	19.1 ± 2.2	20.8 ± 3.3	17.2 ± 3.8

^z^No data recorded.

A total of 160 bulb crates (39.3 cm wide × 59.7 cm long × 17.8 cm tall; 0.23 m^2^) were filled with a soilless medium containing (by volume) 70% peat moss, 21% perlite, and 9% vermiculite (Suremix; Michigan Grower Products Inc., Galesburg, MI, United States). After 14 and 19 d under young-plant photoperiods for the first rep. (29 September 2020) and second rep. (27 September 2021), respectively, 160 witchgrass young plants were randomly selected for transplant from each treatment: 9-, 12-, 13-, 14-, 16-, 18-, and 24-h photoperiods or the 4-h NI (1,280 young plants total). For marigold, 80 young plants were randomly selected for transplant from each treatment: 11-, 13-, 14-, 15-, 16-, and 24-h photoperiods or the 4-h NI (560 young plants total). Eight bulb crates designated for witchgrass seedlings were placed under each photoperiod under both the very low and moderate DLI treatments, and four bulb crates designated for marigold seedlings were placed under each photoperiod under the moderate DLI treatment. Each of the eight or four bulb crates was divided into two or four sections, respectively, yielding 32 sections total per bench (16 sections each for witchgrass and marigold). Five witchgrass or marigold seedlings from one of the aforementioned young-plant treatments were transplanted into a block at a density of 43 or 97 plants per m^2^, respectively. This was repeated randomly across the sections until 10 seedlings from each aforementioned witchgrass or marigold young-plant treatment were transplanted per bench.

One layer of 15 cm supportive netting (HGN32804; Hydrofarm, Petaluma, CA, United States) was positioned ≈15 cm above the bulb crates on each bench. Plants were irrigated as needed with MSU Orchid RO Special [13N-1.3P-12.3K water-soluble fertilizer containing (mg^⋅^L^–1^) 125 nitrogen, 13 phosphorus, 121 potassium, 76 calcium, 19 magnesium, 1.7 iron, 0.4 copper and zinc, 0.9 manganese, 0.2 boron, and 0.2 molybdenum; (GreenCare Fertilizers Inc.)] blended with reverse-osmosis water.

### Data collection and analysis

Ten randomly selected young plants from each treatment were monitored daily for the presence of the first visible flower bud (VB). After 14 or 19 d for reps. 1 and 2, respectively, fully-expanded leaf number, node number, and height from the bottom of the media to the tallest point of the seedling were recorded for these young plants. Additionally, root dry mass (RDM) and shoot dry mass (SDM) were assessed after gently rinsing media from the roots and drying the plant material in an oven for a minimum of 3 d at 70°C.

During the finishing stage, the individual stems of each witchgrass plug were monitored daily for the presence of VB. On this date, the node number below the first VB was recorded. Individual stems of each witchgrass seedling were also monitored daily for the presence of the first open flower (OF) and the date was recorded. On the date of OF, stem length from the media to the tallest point of the most developed stem, branch number, and stem caliper at the thickest point of the stem was recorded with a digital caliper (3-inch carbon fiber digital caliper, General Tools and Instruments, LLC, New York, NY, United States). For witchgrass, the date of harvest (indicated by plants becoming ≥ 50 cm tall with a fully developed panicle) was recorded for the most developed plant in each plug, and for marigold, the date of harvest (indicated by plants becoming ≥ 65 cm tall and terminal blossom 50% open) was recorded for each plant. On the date of harvest, stem length from the media to the tallest point of the inflorescence, stem caliper at the thickest point of the stem, branch number, and the total number of initiated inflorescences were recorded for marigold. Data were analyzed using SAS (version 9.4; SAS Institute, Cary, NC, United States) mixed model procedure (PROC MIXED) for analysis of variance (ANOVA), and means were separated by Tukey’s honest significant difference (HSD) test at *P* ≤ 0.05.

## Results

### Young plant morphology and dry mass

**FIGURE 1:**
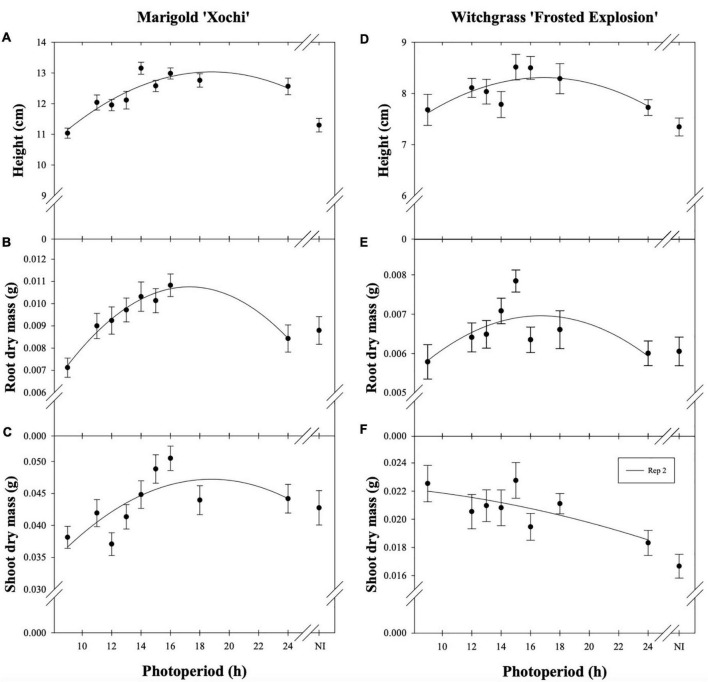
Effect of photoperiod [9, 11, 12, 13, 14, 15, 16, 18, and 24 h, or a 4-h night interruption (NI)] on the height **(A,D)**, root dry mass **(B,E)**, and shoot dry mass **(C,F)** of marigold ‘Xochi’ (*Tagetes erecta*) and witchgrass ‘Frosted Explosion’ (*Panicum capillare*) young plants. Panel **(F)** presents data from replication 2 as trends from replication 1 were not significant. Coefficients are presented in Table 7.

Neither witchgrass nor marigold young plant node or leaf number were influenced by photoperiod (data not reported). Additionally, no plants initiated VBs during the young-plant stage. However, as the young-plant photoperiod increased from 9 to 16 h, the height of witchgrass and marigold increased by 0.8 cm and 1.9 cm, respectively, and then decreased over 16 h (Figures 1A,D). As the photoperiod increased from 9 to 18 h for witchgrass and 10 to 16 h for marigold, RDM increased by up to 14% and 52%, respectively. However, as the photoperiod increased to 24 h, RDM decreased by 9% and 22% for witchgrass and marigold, respectively (Figures 1B,E). The SDM of witchgrass decreased by 41% as the photoperiod increased from 9 to 24 h. In contrast, as the photoperiod increased from 9 to 16 h, the SDM of the marigold increased by up to 32%, after which the SDM decreased by 13% as the photoperiod increased to 24 h (Figures 1C,F).

### Time to visible flower bud

**FIGURE 2:**
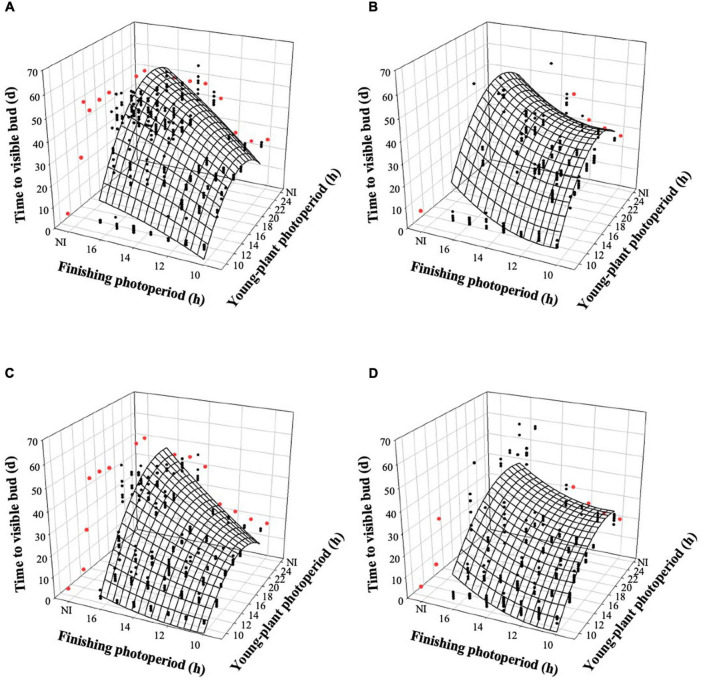
Effects of young-plant photoperiod (9, 12, 13, 14, 16, 18, and 24 h, or a 4-h NI) and finishing photoperiod (10, 11, 12, 13, 14, 15, and 16 h, or a 4-h NI) on time to visible bud of witchgrass ‘Frosted Explosion’ (*Panicum capillare*). Figures represent **(A)** moderate-DLI-grown (≈10 mol^⋅^m^– 2⋅^d^– 1^) cut flowers from replication 1, **(B)** very-low-DLI-grown cut flowers from replication 1, **(C)** moderate-DLI-grown cut flowers from replication 2, and **(D)** very-low-DLI-grown (≈3 mol^⋅^m^– 2⋅^d^– 1^) cut flowers from replication 2. Black circles represent individual data points for sequential photoperiods; red circles represent averages from NI treatments. Model predictions are represented by response surfaces; coefficients are presented in Table 8.

The young-plant and finishing photoperiods interacted to control the time to VB (TVB) of witchgrass (*P* < 0.0001). TVB increased quadratically by 18 or 14 d, for rep. 1 and 2, respectively, when the young-plant photoperiod increased from 9 to 24 h and plants were finished under a 10-h photoperiod and a moderate DLI (Figures 2A,C). This relationship was further accentuated under a longer finishing photoperiod; TVB increased quadratically by an average of 38 d as the young-plant photoperiod increased from 9 to 24 h under a 16-h finishing photoperiod. TVB was also influenced by finishing photoperiod, particularly as the young-plant photoperiod increased. For example, TVB of plants grown under a 9-h young-plant photoperiod was delayed by ≈1 d as the finishing photoperiod increased from 10 to 16 h. However, TVB of plants grown under a 24-h young-plant photoperiod was delayed by ≈23 d as the finishing photoperiod increased from 10 to 16 h. Similar trends, although attenuated, were seen for plants finished under a very low DLI (Figures 2B,C).

**FIGURE 3:**
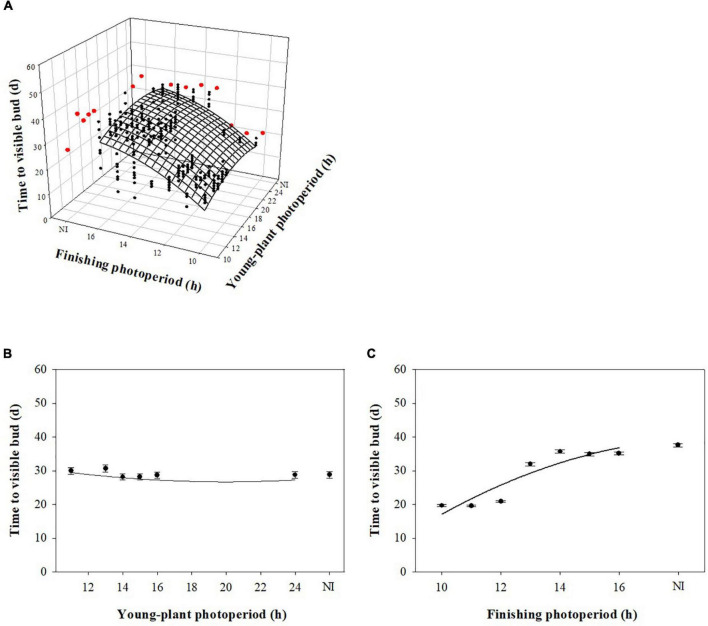
Effect of young-plant photoperiod (11, 13, 14, 15, 16, and 24 h, or a 4-h NI) and/or finishing photoperiod (10, 11, 12, 13, 14, 15, and 16 h, or a 4-h NI) on time to visible bud (TVB) of marigold ‘Xochi’ (*Tagetes erecta*). Figures represent **(A)** the interaction between young-plant and finishing photoperiod on TVB of plants from replication 1, **(B)** the effect of young-plant photoperiod on TVB of plants from replication 2, and **(C)** the effect of finishing photoperiod on TVB of plants from replication 2. In panel **(A)**, black circles represent individual data points for sequential photoperiods; red circles represent averages from NI treatments. Coefficients are presented in Table 9.

Young-plant and finishing photoperiod interacted to influence TVB of marigold during rep. 1 (*P* < 0.0001). However, young-plant photoperiod did not commercially influence TVB and finishing photoperiod had the dominant effect. For instance, TVB of plants finished under 10-h photoperiods increased by only ≈1 d as the young-plant photoperiod increased from 11 to 24 h (Figure 3A). TVB increased by only ≈2 d as the young-plant photoperiod increased from 11 to 24 h when plants were finished under a 16-h photoperiod. In comparison, TVB increased by ≈18 d as the finishing photoperiod increased from 10 to 16 h for plants grown under 11-h young-plant photoperiods. Conversely, young-plant and finishing photoperiod independently influenced TVB of plants grown during the rep. 2 (*P* = 0.23). As the young-plant photoperiod increased from 11 to 24 h, TVB decreased by ≈1 d (Figure 3B). As the finishing photoperiod increased from 10 to 16 h, TVB increased by ≈16 d (Figure 3C).

### Node number below the visible bud

Witchgrass seedlings grown under 9- to 12-h or 9- to 13-h photoperiods during reps. 1 and 2, respectively, developed ≈4 nodes below the first VB regardless of finishing photoperiod or DLI. For plants grown under longer young-plant photoperiods, node number increased proportionally with the finishing photoperiod. Plants grown under 13- (rep. 1) or 14-h (rep. 2) young-plant photoperiods had up to ≈2 more nodes below the first VB as the finishing photoperiod increased from 10 to 16 h, or a 4-h NI, under a moderate DLI. A similar trend was observed for very low DLI grown plants (data not reported). Marigold grown under a 10-h finishing photoperiod formed VBs after a minimum of six nodes had developed, and node count increased up to nine nodes as the finishing photoperiod increased to 16 h (data not reported).

### Time to open flower of witchgrass

**FIGURE 4:**
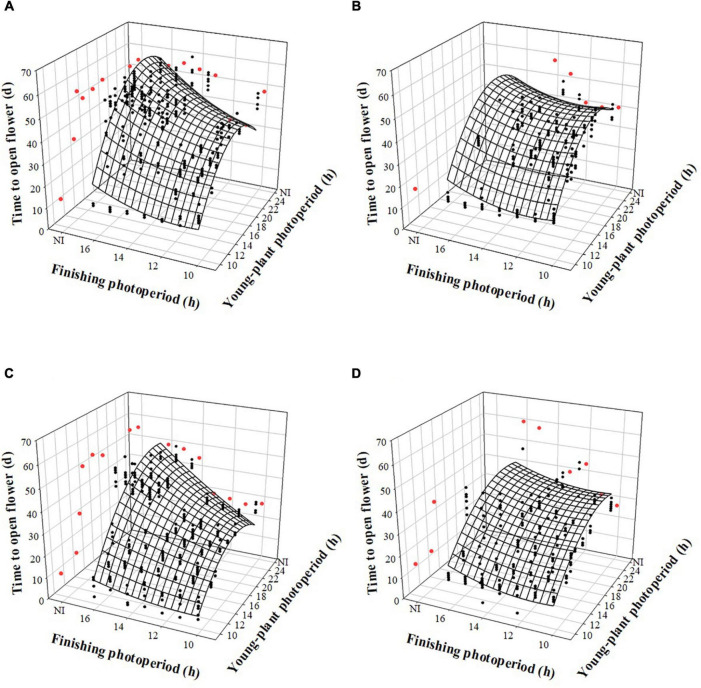
Effects of young-plant photoperiod [9, 12, 13, 14, 16, 18, and 24 h, or a 4-h night interruption (NI)] and finishing photoperiod (10, 11, 12, 13, 14, 15, and 16 h, or a 4-h NI) on time to open flower of witchgrass ‘Frosted Explosion’ (*Panicum capillare*). Figures represent **(A)** moderate-DLI-grown (≈10 mol^⋅^m^– 2⋅^d^– 1^) cut flowers from replication 1, **(B)** very-low-DLI-grown (≈3 mol^⋅^m^– 2⋅^d^– 1^) cut flowers from replication 1, **(C)** moderate-DLI-grown cut flowers from replication 2, and **(D)** very-low-DLI-grown cut flowers from replication 2. Black circles represent individual data points for sequential photoperiods; red circles represent averages from NI treatments. Model predictions are represented by response surfaces; coefficients are presented in Table 8.

The time to open flower (TOF) of witchgrass was influenced by the interaction between the young-plant and finishing photoperiods, following a trend similar to TVB. TOF increased by up to 22 or 15 d, for reps. 1 and 2, respectively, as the young-plant photoperiod increased from 9 to 24 h under a finishing photoperiod of 10 h and a moderate DLI (Figures 4A,C). This effect was stronger under a longer finishing photoperiod. For instance, under a finishing photoperiod of 16 h, TOF increased quadratically by 38 or 34 d, for reps. 1 and 2, respectively, as the young-plant photoperiod increased from 9 to 24 h. The effect of finishing photoperiod on TOF accentuated as the young-plant photoperiod increased. For example, TOF of plants grown under a 12-h young-plant photoperiod was delayed by ≈5 or 1 d as the finishing photoperiod increased from 10 to 16 h for reps. 1 and 2, respectively. However, flowering of plants grown under a 24-h young-plant photoperiod was delayed by ≈9 or 19 d as the finishing photoperiod increased from 10 to 16 h for reps. 1 and 2, respectively. Plants finished under a very low DLI experienced a similar trend, although fewer plants flowered when grown under ≥ 13- (rep. 1) or ≥ 14-h (rep. 2) young-plant photoperiods, or a 4-h NI, and ≥ 13- (rep. 1) or ≥ 14-h (rep. 2) finishing photoperiods or a 4-h NI (Figures 4B,D).

### Witchgrass stem length, caliper, and branch number at open flower

**FIGURE 5:**
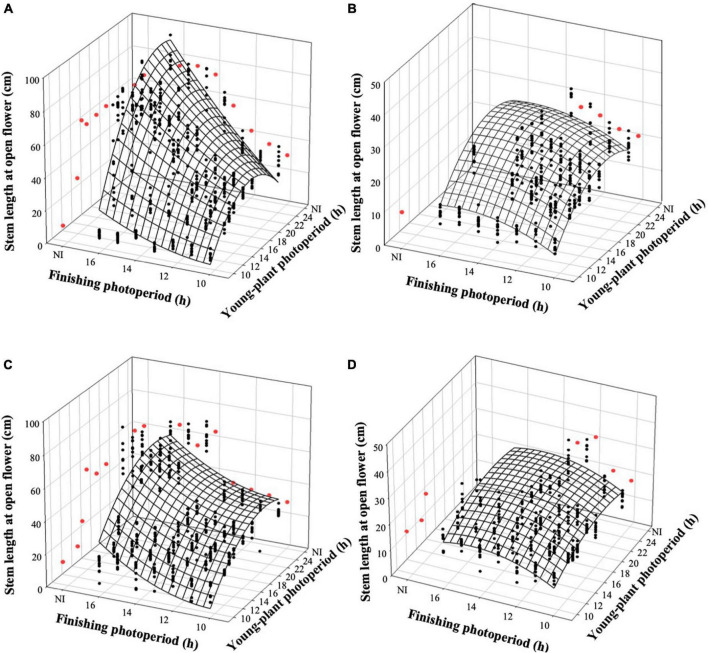
Effects of young-plant photoperiod [9, 12, 13, 14, 16, 18, and 24 h, or a 4-h night interruption (NI)] and finishing photoperiod (10, 11, 12, 13, 14, 15, and 16 h, or a 4-h NI) on stem length of witchgrass ‘Frosted Explosion’ (*Panicum capillare*) at the open flower. Figures represent **(A)** moderate-DLI-grown (≈10 mol^⋅^m^– 2⋅^d^– 1^) cut flowers from replication 1, **(B)** very-low-DLI-grown (≈3 mol^⋅^m^– 2⋅^d^– 1^) cut flowers from replication 1, **(C)** moderate-DLI-grown cut flowers from replication 2, and **(D)** very-low-DLI-grown cut flowers from replication 2. Black circles represent individual data points for sequential photoperiods; red circles represent averages from NI treatments. Model predictions are represented by response surfaces; coefficients are presented in Table 8.

The stem length of witchgrass at OF was proportional to the TOF and was influenced by the interaction of young-plant and finishing photoperiods. As the young-plant photoperiod increased from 9 to 24 h, under a finishing photoperiod of 10 h, stem length increased by an average of 19.5 and 11.0 cm for reps. 1 and 2, respectively (Figures 5A,C). This effect was strengthened as the finishing photoperiod increased; the stem length of plants finished under a 16-h photoperiod and grown under a young-plant photoperiod of 9 h was 71.1 and 42.0 cm shorter than those grown under a 24-h young-plant photoperiod for reps. 1 and 2, respectively. Furthermore, the stem length of witchgrass increased by 1.0 and 2.0 cm for reps. 1 and 2, respectively, when seedlings were grown under 9 h photoperiods and the finished plant photoperiod increased from 10 to 16 h. Conversely, when seedlings were grown under a 24-h young-plant photoperiod and finished under a 16-h photoperiod, stems were 51.6 or 33.0 cm longer than those finished under a 10-h photoperiod for reps. 1 and 2, respectively. Similar trends were seen for the very-low-DLI-grown plants that reached OF, although stem lengths were shorter than the plants finished under the moderate DLI treatment (Figures 5B,D). None of the plants finished under the very low DLI were long enough or developed enough by the end of the study (≈62 d) to be considered harvestable.

Young-plant and finishing photoperiod interacted to influence stem caliper of witchgrass. The stem caliper of plants grown under a 10-h finishing photoperiod was 0.8 or 1.0 mm thicker for reps. 1 and 2, respectively, as the young-plant photoperiod increased from 9 to 24 h (Table 4). This effect was accentuated as the finishing photoperiod increased; the stem caliper of plants finished under a 16-h photoperiod was 3.5 or 4.0 mm thicker when the young-plant photoperiod was 24 h compared to 9 h. Thicker stem calipers were recorded for plants finished under 16-h photoperiods compared to 10-h photoperiods when young plants were grown under 9-h photoperiods. This effect strengthened as the young-plant photoperiod increased. The stem caliper of plants grown under a 24-h young-plant photoperiod was 2.8 or 3.2 mm greater for reps. 1 and 2, respectively, as the finishing photoperiod increased from 10 to 16 h (Table 4). Similar trends, although attenuated, were seen for the very-low-DLI-grown plants that reached OF. However, stem caliper measurements generally ranged only from 0.4 to 2.3 mm for rep. 1 and from 1.2 to 2.5 mm for rep. 2. Plants grown under a moderate DLI had one to three branches at OF, regardless of young-plant or finishing photoperiod, while those grown under a very low DLI had none to two branches at OF.

**TABLE 4:** Effects of young-plant photoperiod (9, 12, 13, 14, 16, 18, and 24 h, or a 4-h NI) and finishing photoperiod (10, 11, 12, 13, 14, 15, and 16 h, or a 4-h NI) on stem caliper (mm) of witchgrass ‘Frosted Explosion’ (*Panicum capillare*) at the open flower.

	Young-plant photoperiod (h)							
Finishing photoperiod (h)	9	12	13	14	16	18	24	NI
*Rep. 1*								
10	0.61	1.05	1.31	1.17	1.68	1.41	1.43	1.41
11	0.69	0.97	2.00	2.06	2.35	2.35	2.41	2.08
12	0.74	1.61	2.36	2.26	2.58	2.50	2.61	2.70
13	0.58	2.66	3.77	4.10	3.59	4.01	4.11	3.64
14	0.82	3.06	3.96	4.62	4.30	4.19	3.73	4.05
15	0.63	3.22	3.98	4.61	3.91	4.35	4.43	3.94
16	0.67	3.59	4.45	4.40	4.12	4.38	4.25	3.89
NI	0.88	3.37	3.89	4.38	3.96	3.93	4.23	4.04
*Rep. 2*								
10	1.42	1.74	2.34	2.31	2.33	2.63	2.39	2.39
11	1.53	1.82	2.40	2.54	2.61	2.66	2.52	2.53
12	1.65	1.74	2.43	2.26	2.80	2.78	2.68	2.46
13	1.53	1.89	2.58	3.16	3.25	3.47	3.46	3.46
14	1.60	1.88	3.37	5.30	5.61	5.10	5.29	4.88
15	1.64	1.97	3.20	4.83	5.56	5.18	5.41	5.17
16	1.56	1.95	3.63	5.75	5.75	5.91	5.61	5.40
NI	1.73	1.87	3.86	5.28	5.91	5.41	5.62	5.28

Cut flowers were finished under a moderate DLI of ≈ 10 mol^⋅^m^–2⋅^d^–1^.

### Time to harvest

During rep. 1, witchgrass stems were only harvestable when seedlings were grown under a photoperiod ≥ 13 h and finished under a photoperiod ≥ 13 h and a moderate DLI. Plants finished under photoperiods < 13 h flowered prematurely and were unmarketable. Generally, plants grown under 13-h young-plant and finishing photoperiods became harvestable the fastest, whereas those grown under a NI during the young-plant and finishing stages were the slowest to reach harvest (Table 5). During rep. 2, plants grown under young-plant photoperiods < 14 h and finishing photoperiods < 14 h flowered prematurely. All harvestable plants were harvested within a 10- to 13-d timeframe, depending on reps. (Table 5). Plants finished under a very low DLI did not yield harvestable stems.

**TABLE 5:** Effects of young-plant photoperiod (9, 12, 13, 14, 16, 18, and 24 h, or a 4-h NI) and finishing photoperiod (10, 11, 12, 13, 14, 15, and 16 h, or a 4-h NI) on time to harvest (d) from the date of transplant of witchgrass ‘Frosted Explosion’ (*Panicum capillare*) grown under a moderate DLI of ≈ 10 mol^⋅^m^–2⋅^d^–1^.

	Young-plant photoperiod (h)							
Finishing photoperiod (h)	9	12	13	14	16	18	24	NI
*Rep. 1*								
10	–*^z^*	–*^z^*	–*^z^*	–*^z^*	–*^z^*	–*^z^*	–*^z^*	–*^z^*
11	–*^z^*	–*^z^*	–*^z^*	–*^z^*	–*^z^*	–*^z^*	–*^z^*	–*^z^*
12	–*^z^*	–*^z^*	–*^z^*	–*^z^*	–*^z^*	–*^z^*	–*^z^*	–*^z^*
13	–*^z^*	–*^z^*	53	53	53	51	53	54
14	–*^z^*	–*^z^*	54	53	53	54	56	56
15	–*^z^*	–*^z^*	58	57	56	58	53	55
16	–*^z^*	–*^z^*	55	55	57	58	58	55
NI	–*^z^*	–*^z^*	60	55	55	56	56	58
*Rep. 2*								
10	–*^z^*	–*^z^*	–*^z^*	–*^z^*	–*^z^*	–*^z^*	–*^z^*	–*^z^*
11	–*^z^*	–*^z^*	–*^z^*	–*^z^*	–*^z^*	–*^z^*	–*^z^*	–*^z^*
12	–*^z^*	–*^z^*	–*^z^*	–*^z^*	–*^z^*	–*^z^*	–*^z^*	–*^z^*
13	–*^z^*	–*^z^*	–*^z^*	–*^z^*	–*^z^*	–*^z^*	–*^z^*	–*^z^*
14	–*^z^*	–*^z^*	–*^z^*	49	48	47	47	48
15	–*^z^*	–*^z^*	–*^z^*	51	49	53	51	51
16	–*^z^*	–*^z^*	–*^z^*	52	50	48	48	51
NI	–*^z^*	–*^z^*	–*^z^*	60	58	57	57	60

^z^No harvestable stems by the end of study.

Only marigolds grown under 10- to 12-h finishing photoperiods were harvestable by the end of the study (≈50 d). However, up to 29% and 107% more stems were harvested under 11- and 12-h finishing photoperiods, respectively, compared to the 10-h photoperiod (data not reported). Time to harvest of marigold finished under 10-, 11-, and 12-h photoperiods ranged from 40 to 48 d after transplant (Table 6).

**TABLE 6:** Effects of young-plant photoperiod (11, 13, 14, 15, 16, or 24 h, or a 4-h NI) and finishing photoperiod (10, 11, 12, 13, 14, 15, and 16 h, or a 4-h NI) on time to harvest (d) of marigold ‘Xochi’ (*Tagetes erecta*).

	Young-plant photoperiod (h)						
Finishing photoperiod (h)	11	13	14	15	16	24	NI
*Rep. 1*							
10	46	48	43	47	44	44	44
11	41	43	43	44	43	45	40
12	43	46	42	43	46	46	45
13	–*^z^*	–*^z^*	–*^z^*	–*^z^*	–*^z^*	–*^z^*	–*^z^*
14	–*^z^*	–*^z^*	–*^z^*	–*^z^*	–*^z^*	–*^z^*	–*^z^*
15	–*^z^*	–*^z^*	–*^z^*	–*^z^*	–*^z^*	–*^z^*	–*^z^*
16	–*^z^*	–*^z^*	–*^z^*	–*^z^*	–*^z^*	–*^z^*	–*^z^*
NI	–*^z^*	–*^z^*	–*^z^*	–*^z^*	–*^z^*	–*^z^*	–*^z^*
*Rep. 2*							
10	45	44	42	45	42	43	43
11	42	41	41	42	44	44	42
12	44	44	41	41	42	42	41
13	–*^z^*	–*^z^*	–*^z^*	–*^z^*	–*^z^*	–*^z^*	–*^z^*
14	–*^z^*	–*^z^*	–*^z^*	–*^z^*	–*^z^*	–*^z^*	–*^z^*
15	–*^z^*	–*^z^*	–*^z^*	–*^z^*	–*^z^*	–*^z^*	–*^z^*
16	–*^z^*	–*^z^*	–*^z^*	–*^z^*	–*^z^*	–*^z^*	–*^z^*
NI	–*^z^*	–*^z^*	–*^z^*	–*^z^*	–*^z^*	–*^z^*	–*^z^*

^z^No harvestable stems by the end of study.

**TABLE 7:** Regression analysis equations and r^2^ or R^2^ for height, root dry mass, and shoot dry mass in response to photoperiod (P; 9-, 11-, 12-, 13-, 14-, 15-, 16-, 18-, 24-h photoperiods or a 4-h NI) of marigold ‘Xochi’ (*Tagetes erecta*) or witchgrass ‘Frosted Explosion’ (*Panicum capillare*).

Parameter	y0	a	b	R^2^ or r^2^
	**Marigold ‘Xochi’**			
Height (cm)	6.09*^z^*	0.74	−0.02	0.240
Root dry mass (g)	−0.00	0.00	−5.14E-05	0.174
Shoot dry mass (g)	−0.01	0.00	0.00	0.102
	**Witchgrass ‘Frosted Explosion’**			
Height (cm)	5.14	0.38	−0.01	0.049
Root dry mass (g)	0.00	0.00	−1.92E-05	0.070
Shoot dry mass (g)	0.02	−5.27E-06	-6.83E-06	0.075

^z^Coefficients for model equations were used to generate Figure 1A through Figure 1F.

All models are in the form of: f = y0 + a*P + b*P^2^.

**TABLE 8:** Regression analysis equations and r^2^ or R^2^ for time to visible bud; time to open flower; and stem length at the open flower in response to young-plant photoperiod (YP; 9-, 12-, 13-, 14-, 16-, 18-, or 24-h, or a 4-h night interruption; NI) and finishing photoperiod (FP; 10-, 11-, 12-, 13-, 15-, or 16-h, or a 4-h NI) of witchgrass ‘Frosted Explosion’ (*Panicum capillare*).

Parameter	y0	a	b	c	d	e	R^2^ or r^2^
**Time to visible bud (d)**							
*Rep. 1 Moderate DLI*	−100.65*^z^*	7.86	5.09	−0.29	−0.18	0.25	0.774
*Rep. 1 Very Low DLI*	*^y^*	11.17	−13.33	−0.29	0.64		0.638
*Rep. 2 Moderate DLI*		4.56	−7.53	−0.23	0.20	0.36	0.761
*Rep. 2 Very Low DLI*		7.67	−10.27	−0.18	0.49		0.589
**Time to open flower (d)**							
*Rep. 1 Moderate DLI*		9.05	−9.41	−0.30	0.36	0.19	0.652
*Rep. 1 Very Low DLI*		10.26	−9.07	−0.27	0.40		0.745
*Rep. 2 Moderate DLI*		4.72	−5.98	−0.21	0.16	0.29	0.742
*Rep. 2 Very Low DLI*		6.70	−6.39	−0.16	0.29		0.585
**Stem length at open flower (cm)**							
*Rep. 1 Moderate DLI*		8.17	−13.32	−0.41	0.43	0.62	0.783
*Rep. 1 Very Low DLI*	−96.37	5.42	9.84	−0.14	−0.32		0.686
*Rep. 2 Moderate DLI*		10.08	−13.50	−0.24	0.70		0.589
*Rep. 2 Very Low DLI*	−90.87	2.91	12.44	−0.07	−0.43		0.478

^z^Coefficients for model equations were used to generate Figure 2 through Figure 4.

^y^Blank cells = 0.

All models are in the form of: f = y0 + a*YP + b*FP + c*YP^2^ + d*FP^2^ + e*(YP*FP).

Cut flowers were finished under a moderate DLI of ≈ 10 mol^⋅^m^–2⋅^d^–1^ or a very low DLI of ≈ 3 mol^⋅^m^–2⋅^d^–1^.

**TABLE 9:** Regression analysis equations and r^2^ or R^2^ for time to visible bud in response to young-plant photoperiod (11, 13, 14, 15, 16, 24 h, or a 4-h NI) and/or finishing photoperiod (10, 11, 12, 13, 14, 15, 16 h, or a 4-h NI) of marigold ‘Xochi’ (*Tagetes erecta*).

Figure	y0	a	b	c	d	R^2^ or r^2^
*3A*	−94.22*^z^*	3.26	11.72	0.09	−0.33	0.797
*3B*	40.34	−1.36	0.03	*^y^*	*^y^*	0.098
*3C*	−57.28	10.05	−0.26	*^y^*	*^y^*	0.836

^z^Coefficients for model equations were used to generate Figure 3A through Figure 3C.

^y^Blank cells = 0.

Models 3A is in the form of: f = y0 + a*YP + b*FP + c*YP^2^ + d*FP^2^ and models 3B and 3C are in the form of: f = y0 + a*P + b*P^2^

### Marigold stem length, caliper, branch, and inflorescence number at harvest

Finishing photoperiod had the dominant effect on marigold stem length at harvest, and young-plant and finishing photoperiods did not interact to influence the length of the stems that became harvestable. As the finishing photoperiod increased from 10 to 12 h, stem length at harvest increased from 70 to 74 cm. While plants grown under photoperiods > 12 h were not harvestable at the end of the study, they were at least 65 cm long, regardless of finishing photoperiod (data not reported), indicating the potential for all stems to eventually reach marketability. Stem caliper and branch and inflorescence numbers at harvest of plants grown under finishing photoperiods of 10 to 12 h were not significantly different (data not reported).

## Discussion

The results of this study further support that photoperiod manipulation during the young-plant and finishing stages, in addition to maintaining or increasing the DLI, can aid in producing high-quality specialty cut flowers while reducing crop time. Growers can manipulate these environmental parameters to improve finished cut flower quality and reduce the time to harvest. These techniques are particularly useful when the natural photoperiod is not conducive to the photoperiodic responses of the crop to be grown, or when solar radiation is limiting.

When grown under inductive conditions, specialty cut flowers can flower prematurely with unmarketable stem lengths (Dole and Warner, 2017). Witchgrass demonstrated this phenomenon, which is consistent with several other publications on photoperiodic lighting of SDPs. Plants in this study grown under 9- to 12-h (rep. 1) or 9- to 13-h (rep. 2) young-plant photoperiods flowered prematurely, regardless of finishing photoperiod. Premature flowering was also seen for witchgrass finished under 10- to 12-h (rep. 1) or 10- to 13-h (rep. 2) finishing photoperiods, regardless of young-plant photoperiod. These findings indicate that the critical photoperiod of witchgrass ‘Frosted Explosion’ is 12 to 13 h. During rep. 2, the stem length of plants grown under photoperiods ≤ 13 h were only 10 to 32 cm long at OF, which is below the market minimum of 50 cm (BloomStudios, 2020, personal communication). Similarly, marigolds finished under inductive photoperiods were shorter than those under non-inductive photoperiods, although all treatments would have yielded marketable stem lengths upon flowering.

Jensen et al. (2012) reported similar trends to witchgrass after investigating the photoperiodic response of Amur silvergrass (*Miscanthus sacchariflorus*), a grass used as a biofuel. The flowering of *Miscanthus* was delayed by 83 d under LDs (15.3-h photoperiods) compared to gradually decreasing SDs (15.3-h photoperiods for 21 d, followed by 119 d of a decreasing photoperiod consistent with that at 34.1°N), designating it as a facultative SDP. Plants grown under LDs accumulated ≈52% more biomass (stem and leaf tissue) compared to plants grown under SDs, aligning with the witchgrass stem caliper increase in the present study. The authors hypothesized that stem length under LDs would likely have been longer than those grown under SDs if their experiment ran longer, as *Miscanthus* exhibits rapid stem elongation during the emergence of flag leaves, which is ≈18 d after floral initiation (Jensen et al., 2012). However, the experiment was terminated before LD-mediated plant elongation would have occurred. During rep. 2 of the present study, witchgrass finished under a 14-h LD were up to 224% longer at OF than plants finished under a 13-h SD, suggesting a similar stem elongation response.

In the present study, witchgrass may have a similar sensitivity to inductive photoperiods as celosia (*Celosia argentea* var. *plumosa*) during the young-plant stage. Warner (2009) reported that the SDP celosia ‘Gloria Scarlet’ overcame juvenility and perceived inductive treatments 9 to 12 d after cotyledon emergence. Plants were exposed to 12 9-h SDs before cotyledon emergence, then placed under a 4-h NI from 2200 to 0200 h, and had ≈7 fewer nodes below the terminal inflorescence than plants grown under continuous LDs. Moreover, plants exposed to 12 SDs at the beginning 3 d after cotyledon emergence had a similar node number below the terminal inflorescence compared to those under continual SDs (Warner, 2009). This could explain why plants grown under inductive conditions during the young-plant stage flowered prematurely, even when transferred to non-inductive finishing conditions. Further experimentation to determine when witchgrass begins reproductive development may be necessary. However, growers should avoid premature flower-inductive conditions to ensure proper market specifications are met.

TVB of marigold was negligibly influenced by young-plant photoperiod, suggesting marigold was not induced to flower during the first 2 weeks of growth. This is inconsistent with Warner (2006), who identified the photoperiod-sensitive stages of the SDPs cosmos ‘Sonata White’ (*Cosmos bipinnatus*) and signet marigold ‘Tangerine Gem’ (*Tagetes tenuifolia*). It was reported that both species were receptive to inductive conditions after 1 to 2 leaf pairs had unfolded, with five 9-h SDs delivered after cotyledon emergence promoting flowering of cosmos by 23 d compared to a constant 4-h NI treatment. Furthermore, marigolds exposed to 5 SDs after cotyledon emergence flowered ≈10 d faster than plants grown under continual LDs (Warner, 2006).

Although the variables interacted, finishing photoperiod had a greater effect on TVB of marigold than young-plant photoperiod. Plants that finished under 10- to 12-h photoperiods had faster TVB than those finished under photoperiods ≥ 13 h (Figure 3C). Therefore, the critical photoperiod of marigold ‘Xochi’ was determined to be ≈12 h. Marigold’s flowering responses align with other studies detailing photoperiodic responses of SDPs (Park et al., 2013; Kang et al., 2019). TVB of zinnia ‘Dream Land’ decreased by 10 d when finished under 9-h SDs instead of 4-h NI (Park et al., 2013). Similarly, Kang et al. (2019) demonstrated that 8-h SDs promoted flowering of kalanchoe ‘Lipstick’ (*Kalanchoe blossfeldiana*), while 16-h photoperiods or 4-h NIs inhibited flowering, regardless of NI TPFDs. Unlike marigold ‘Xochi’, kalanchoe ‘Lipstick’ was ≈45 or 33% taller under SDs compared to plants grown under a 16-h LD or a 4-h NI, respectively (Kang et al., 2019). However, this may have been due to the absence of stem elongation associated with flowering, as plants grown under LDs or NIs did not transition from vegetative to reproductive growth.

Absorbed radiation is the driving force for photosynthesis and subsequent plant growth and development. Thus, SL must be utilized when solar radiation is limited to produce high-quality cut flowers year-round. SL had a substantial effect on the growth and development of witchgrass. TVB and TOF were hastened for plants grown under a moderate DLI compared to those grown under a very low DLI. Similarly, Faust et al. (2005) reported that the time to flower of vinca ‘Pacific Lilac’ (*Catharanthus roseus*) and zinnia ‘Dreamland Rose’ decreased by 3 and 10 d, respectively, when grown under a DLI of 43 mol^⋅^m^–2⋅^d^–1^ compared to 5 mol^⋅^m^–2⋅^d^–1^. In a separate study, jasmine tobacco ‘Domino White’ (*Nicotiana alata* Link and Otto) and helipterum (*Helipterum roseum* Hook.) flowered 17 and 6 d faster, respectively, when grown under SL providing 50 μmol^⋅^m^–2⋅^s^–1^ for 18 h compared to those grown without SL (Erwin and Warner, 2002).

One hundred percent of witchgrass plants grown under young-plant photoperiods between 13 and 24 h or a 4-h NI (rep. 1) or 14 and 24 h or a 4-h NI (rep. 2), and finished under photoperiods ≥ 13 h, or a 4-h NI (rep. 1) or ≥ 14 h, or a 4-h NI (rep. 2), and a moderate DLI yielded harvestable stems. Conversely, no plants finished under a low DLI-yielded harvestable stems. Likewise, Furufuji et al. (2014) reported that cut rose ‘Tint’ (*Rosa* spp.) yield was 101% higher when grown with a supplemental DLI of 5.8 mol^⋅^m^–2⋅^d^–1^ compared to those grown without SL. Moreover, greenhouse-grown lisianthus ‘Echo Champagne’ and ‘Rosita White’ (*Eustoma* spp.) produced 12 and 2 more stems per m^2^, respectively, when grown under 67% shade compared to 88% shade for 5 weeks (Lugasi-Ben-Hamo et al., 2010). Furthermore, witchgrass stem length, caliper, and branch number improved when grown under moderate DLIs. Similarly, Torres and Lopez (2011) found that stem caliper of yellow trumpet bush ‘Mayan Gold’ (*Tecoma stans*) seedlings increased by 133% as the DLI increased from 0.8 to 25.2 mol^⋅^m^–2⋅^d^–1^. In another study, stem caliper and height of mountain spike speedwell (*Veronica rotunda* var. *subintegra*) increased by 110% and 77%, respectively, as the DLI increased from 3.6 to 18.3 mol^⋅^m^–2⋅^d^–1^ (Lim et al., 2022).

In conclusion, the present study indicates that high-quality marigold ‘Xochi’ cut flowers can be produced in a timely fashion when young plants are grown under any photoperiod between 11 and 24 h, or a 4-h NI, and finished under a 12-h photoperiod, as stem yield was highest under this finishing photoperiod compared to 10- or 11-h finishing photoperiods. The interactions between young-plant and finishing photoperiods were not commercially impactful for the stems of marigold that reached VB or were harvestable. While marigolds finished under photoperiods > 12 h did not reach harvestability during the study, they all developed flower buds and were likely to have become harvestable, after a delay, compared to marigolds finished under photoperiods < 13 h. As such, the influence of young-plant and finishing photoperiod was not empirically quantified for these plants and could be investigated further in another study. Moreover, high-quality witchgrass ‘Frosted Explosion’ cut flowers can be grown under any photoperiod between 14 and 24 h, or a 4-h NI, during the young-plant stage, and finished under photoperiods equal to or greater than 14-h, or a 4-h NI, to prevent premature flowering and subsequent inferior quality. While these photoperiods yielded cut flowers of similar thickness, 16-h photoperiods can be maintained to produce longer witchgrass stems. Witchgrass should be grown under at least a moderate DLI of ≥ 10 mol^⋅^m^–2⋅^d^–1^ during the finishing stage to produce cut flowers with sufficient stem lengths and calipers for market. Growers once limited to producing witchgrass and marigold outdoors or in high tunnels during warm and temperate seasons may use these recommendations to produce these varieties in greenhouses during the winter and early spring, allowing for consistent production.

## Data availability statement

The raw data supporting the conclusions of this article will be made available by the authors, without undue reservation.

## Author contributions

CS and RL conceptualized and designed the study. CS performed the experiments, conducted data analysis, and prepared the manuscript. RL obtained funding and revised the manuscript. Both authors contributed to the article and approved the submitted version.
